# Integrinβ-1 in disorders and cancers: molecular mechanisms and therapeutic targets

**DOI:** 10.1186/s12964-023-01338-3

**Published:** 2024-01-26

**Authors:** Chen Su, Jie Mo, Shuilin Dong, Zhibin Liao, Bixiang Zhang, Peng Zhu

**Affiliations:** 1 Hepatic Surgery Center, Tongji Hospital, Tongji Medical College, Huazhong University of Science and Technology, 1095 Jiefang Avenue, Wuhan, 430030 Hubei People’s Republic of China; 2 Hubei Key Laboratory of Hepato-Pancreato-Biliary Diseases, Wuhan, Hubei People’s Republic of China; 3 Key Laboratory of Organ Transplantation, Ministry of Education, Wuhan, Hubei People’s Republic of China; 4 Key Laboratory of Organ Transplantation, National Health Commission, Wuhan, Hubei People’s Republic of China; 5 Key Laboratory of Organ Transplantation, Chinese Academy of Medical Sciences, Wuhan, Hubei People’s Republic of China

**Keywords:** Integrinβ-1, Drug target, Cancer metastasis

## Abstract

Integrinβ-1 (ITGB1) is a crucial member of the transmembrane glycoprotein signaling receptor family and is also central to the integrin family. It forms heterodimers with other ligands, participates in intracellular signaling and controls a variety of cellular processes, such as angiogenesis and the growth of neurons; because of its role in bidirectional signaling regulation both inside and outside the membrane, ITGB1 must interact with a multitude of substances, so a variety of interfering factors can affect ITGB1 and lead to changes in its function. Over the past 20 years, many studies have confirmed a clear causal relationship between ITGB1 dysregulation and cancer development and progression in a wide range of benign diseases and solid tumor types, which may imply that ITGB1 is a prognostic biomarker and a therapeutic target for cancer treatment that warrants further investigation. This review summarizes the biological roles of ITGB1 in benign diseases and cancers, and compiles the current status of ITGB1 function and therapy in various aspects of tumorigenesis and progression. Finally, future research directions and application prospects of ITGB1 are suggested.

Video Abstract

**Supplementary Information:**

The online version contains supplementary material available at 10.1186/s12964-023-01338-3.

## Background

**Fig. 1:**
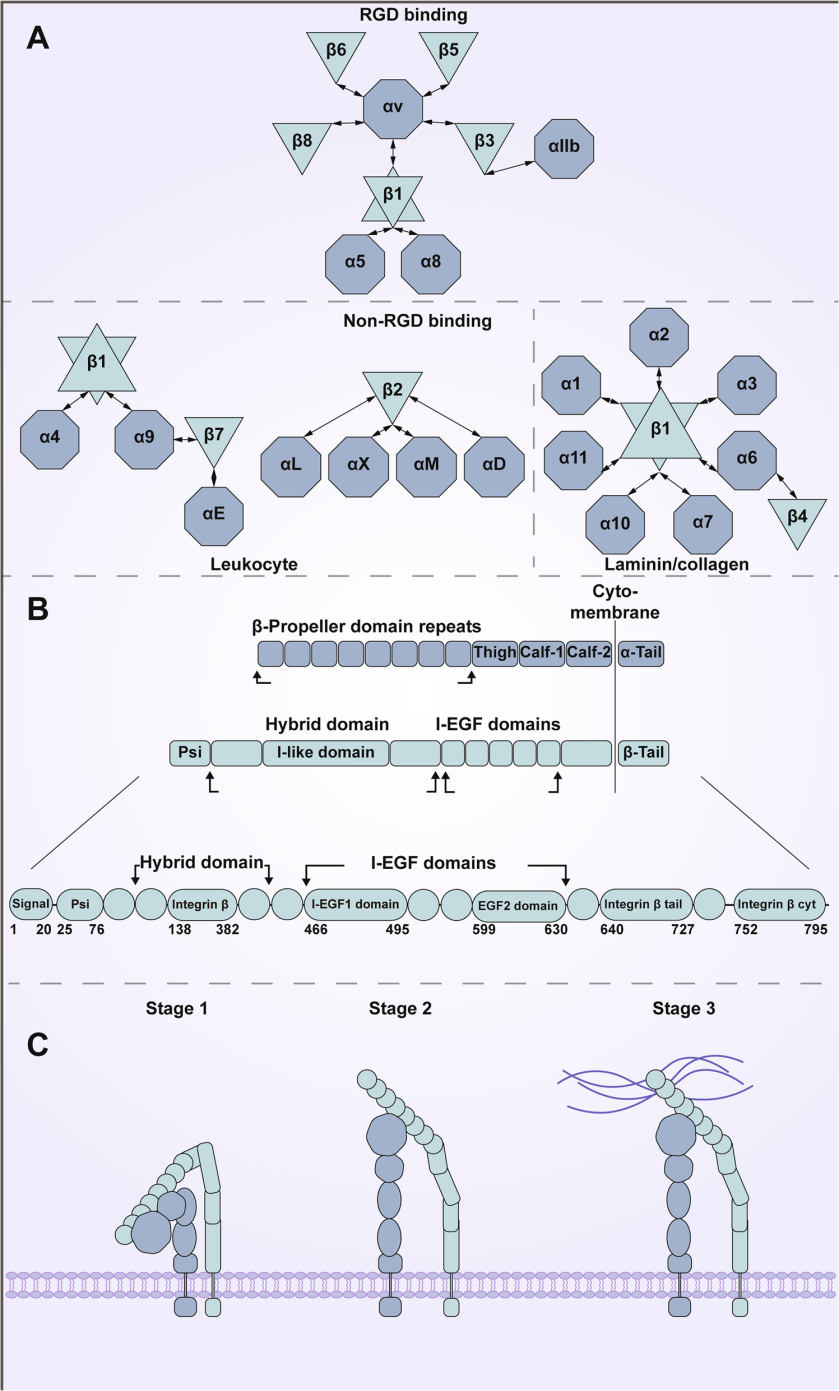
The structure and functions of ITGB1. **A** The α- and β-subunits of the integrin family have 18 and 8 different isoforms, respectively, which are combined with each other to produce 24 heterodimers. The first eight combinations (αvβ1, αvβ3, αvβ5, αvβ6, αvβ8, αIIIbβ3, α5β1, and α8β1) all recognize the amino acid binding pattern RGD in their endogenous ligands and are referred to as the RGD binding family. The second is a combination of integrins (α4β1, α4β7, α9β7, αEβ7, αLβ2, αDβ2, αMβ2, and αXβ2) that can identify the short peptide sequence Leu-Asp-Val and are expressed on leukocytes. In addition, a third combination (α1β1, α2β1, α3β1, α6β1, α7β1, α10β1, α11β1, α6β4) can bind to collagen or laminin. **B** Half of the 18 α-subunits (α1, α2, α10, α11, αD, αL, αM, αX, and αE subunits) have an additional 200 aa of I-domain in the β-propeller domain. The I-like domain is a metal ion-dependent adhesion site (MIDAS). β-subunits usually include a PSI domain, a hybrid domain with an I-like domain (βI), an integrated I-EGF domain, and a β-tail domain. **C** Integrin activation can be divided into three stages: in the first stage, the extracellular segment of integrins is curved and the cytoplasmic tails of the α and β subunits are closed. In the second stage, the TM and extracellular structural domains of the β integrin are forced to unfold upon binding to the adaptor proteins Talin and/or Kindlin, an integrin-binding cofactor. This creates an accessible ligand-binding pocket (poor affinity). In the third stage, when the integrin contacts the ECM, its extracellular segment straightens, while the intracellular segment separates further in the tail. This conformational change helps to regulate the correlation between receptors, cytoskeletal proteins and signal transducers (with high affinity)

Since their discovery in the 1980s, integrins have been developed into cell adhesion transmembrane receptors that act as extracellular matrix (ECM)-cytoskeletal linkers and transmit biochemical and mechanical signals between cells and their environment in a variety of physiological and pathological conditions [1–3]. Transmembrane glycoprotein receptors known as integrin proteins are ubiquitous heterodimers that serve largely as signaling proteins in mammals [4]. The amino acid (aa) sequences of integrins are evolutionarily conserved and the structure of the various variants can be divided into three parts (a large extracellular domain, a short transmembrane domain, and a short cytoplasmic domain). The extracellular domain of these variants participates in ligand binding. There are 18 and 8 different variations of the α and β-subunits that comprise each integrin, respectively, and they combine to produce 24 known heterodimers. Integrin proteins can be categorized into several families based on the different receptors that can be bound. For example, eight members (αvβ1, αvβ3, αvβ5, αvβ6, αvβ8, αIIIbβ3, α5β1, and α8β1) all recognize the amino acid binding pattern Arg-Gly-Asp (RGD) in their endogenous ligands and are called the RGD binding family. Also, integrin families (α4β1, α4β7, α9β7, αEβ7, αLβ2, αDβ2, αMβ2, and αXβ2) that recognize the short peptide sequence Leu-Asp-Valare present and expressed on leukocytes (Fig. 1A) [5, 6]. In addition, there are integrin families (α1β1, α2β1, α3β1, α6β1, α7β1, α10β1, α11β1, α6β4) that bind to collagen 10 or laminin [7] with a wide range of roles in various diseases [8, 9].

ITGB1 (Integrinβ-1, β1, fibronectin receptor, beta polypeptide, antigen CD29 includes MDF2, MSK12), located on human chromosome 10p11.22, is 57,913 bp in length. it encodes a protein with five isoforms (β1-A, β1-B, β1-C 1, β1-C2, and β1-D), among which, β1-A is the most widely studied, which contains 798 aa (all references to ITGB1 below refer to β1-A of 798aa) (Fig. 1B). Researchers first discussed the idea of the “integrin field” in 1980, marking the beginning of the study of integrins. Small molecule RGD sequence was discovered in 1984, and in 1985, GPIIb/IIIa, a typical integrin fragment, was discovered to be a receptor for fibronectin. The class of proteins containing the aforementioned common segment was not given the name “integrins” until 1986. The integrin family’s numerous members were subsequently discovered. All 24 heterodimers were not discovered until 2000. Although ITGB1 was not found on its own, as the nucleus of the integrin family, it has a unique significance. As the first of the β-subunits in the integrin family, it is the most widely distributed and can bind to the largest number of α-subunits, with 12 of the 18 species forming different integrin units therewith. Among them, ITGB1 forms RGD binding with αv, α5, and α8; Leu-Asp-Val forms binding with α4 and α9, as expressed on leukocytes; and complexes with α1, α2, α3, α6, α7, α10, and α11, which are widely expressed on various cells and involved in the recognition of collagen and laminin. It has been proved that integrins with ITGB1 as a subunit are involved in signaling pathway transduction in various cells to maintain normal physiological functions, and in tumor cells, they are involved in maintaining cell stemness and promoting tumor cells to develop invasion and metastasis or chemo-radiotherapy resistance. In summary, ITGB1 as an important subunit of integrin protein, ITGB1 can bind to many ligands to perform different functions.

The molecular structure and numerous functions of ITGB1 were identified in this review. Furthermore, the molecular mechanisms involved in its function in creating cancer medication resistance were discussed. This research summarizes and discusses the pattern of expression of a potent oncogenic receptor protein with clinical importance in diverse malignancies. Lastly, we discussed the effects of ITGB1 on many characteristics of cancer cells and its potential utility as a diagnostic biomarker and a therapeutic target for additional translational research.

## Structure, and function of integrins

### Structure of integrins

Returning our attention to heterodimers, the β-propeller domain of half of the 18 α-subunits (subunits α1, α2, α10, α11, αD, αL, αM, αX, and αE) has an extra 200 aa of the I-domain added thereto [10]. The I-domain, which serves as a key binding site for ligands such as collagen and certain laminins, is a metal ion-dependent adhesion site (MIDAS, a metal ion-dependent adhesion site, found in the I-domain, serves as a key binding site for ligands such as collagen and certain laminins) [11]. Akin to the α-subunit, the β-subunit typically comprises a plexin-sempahorin-integrin (PSI) domain, a hybrid domain (βI) with an I-like domain, an integrin epidermal growth factor-like (I-EGF) domain, and a β-tail domain. By interacting with the β-propeller domain in nine additional α-integrins (α3, α4, α5, α6, α7, α8, α9, αv, and αIIb) that lack an αI-domain, it contributes to ligand binding [12]. The transmembrane helical domains (TMDs) of the α and β subunits are identical, and their connection is necessary for the activation of integrins (Fig. 1B).

Integrin and intercellular protein interactions take place at the nuclear center of the cytoplasmic tail of integrins. The α and β cytoplasmic tails are typically shorter than 75aa, except for the β4 tail, which is about 1000 aa long. In particular, the β4 integrin binds to intermediate filaments rather than the actin cytoskeleton, in contrast to other integrins [13]. The most common sequence found in integrin β tails is NPxY/F, which allows for binding to proteins that contain a phosphotyrosine binding domain, such as Talin (a high molecular weight cytoskeletal protein concentrated in the cell–matrix contact region). Importantly, the cytoplasmic tails of the β-subunits are surprisingly alike, although the tails of the α-subunits are largely variable save for a conserved GFFKR motif around the TM region.

### Functions of integrins

Integrins function as adhesion receptors and have a remarkable capacity to transmit signals across the plasma membrane in either direction of the cell membrane [14]. This capability — known as “intracellular to extracellular” and “extracellular to intracellular” [15], — is dependent on integrin “intracellular domains” interactions with the cytoskeleton or their ability to bind to extracellular ligands. Integrins allow human cells to react to changes in the extracellular environment by signaling from outside to inside as well as to have an impact on the extracellular environment. When the ligand attaches to the receptor, signals from the outside of the cell cascade into the cell, causing changes in gene expression levels, cell polarization, and the ability of the cell to survive and proliferate [16]. However, intracellular activation signals such as those associated with Talin are also present [17]. Integrins may adopt a high-affinity state and bind to extracellular ligands more readily when they are bound to the cytoplasmic tail of the β-subunit, which promotes cell motility and the building and remodeling of the extracellular matrix (ECM) [18]. As a three-dimensional macromolecular network without cells, the ECM consists mainly of an interconnected system of fibrillar and non-protofibrillar collagen, elastic fibers, and glycosaminoglycan-containing non-collagenous glycoproteins (hyaluronic acid and proteoglycans) [19]. The function of the ECM is mainly to maintain tissue integrity, and its loss of control during disease processes can alter its composition and morphology, thus leading to the development of various diseases.

Previous studies show that integrin activation goes through three phases, each of which involves a distinct conformation change: Phase 1: bend-closed (inactive), Phase 2: extended-closed (poor affinity), and Phase 3: extended-open (high affinity) [20, 21]. In the first phase, the extracellular segments of integrins were curved and the cytoplasmic tails of α and β subunits were closed together. The inactive conformation was further stabilized by the interaction between the α and β tails [21]. In the second phase, the TM and extracellular domains of the β integrin were forced to unfold upon binding to the adaptor proteins Talin and/or Kindlin (an integrin-binding coactivator). This created an accessible ligand-binding pocket (poor affinity) [20, 21]. The extracellular segment of the integrin straightens when it meets the ECM, while the intracellular segment separates further at the tail. This conformational change helps regulate connections between the receptor, cytoskeletal proteins, and signal transducers (with high affinity). In turn, these interactions stimulate the development of potent focal adhesions by increasing ligand binding affinity and causing the aggregation of other activated integrins [22]. This adhesion complex uses an indirect integrin-actin coupling (for example, α6β4 integrin to intermediate filaments) to connect the intracellular cytoskeleton to the basement membrane. Significantly, mechanical forces support the cytoskeleton-ECM connection by strengthening it and attracting more signaling proteins that activate integrins (Fig. 1C) [23].

Since integrins are transmembrane proteins without enzymatic activity, they work by attaching to nearby receptors and intracellular proteins to transmit mechanical or chemical signals to the interior of the cell, ultimately affecting how the cell functions. Integrins function as a mechano-sensor and force transducer through this bidirectional linkage, and they also coordinate the actin cytoskeleton to modulate a variety of crucial biological processes, including cell adhesion, migration, proliferation, differentiation, and apoptosis, all of which are frequently dysregulated in cancers. The integrin-mediated adhesion complex, also known as focal adhesion or adhesome, contains approximately 150 adhesion proteins [20, 24]. Therefore, the downstream signaling of an activated integrin (*i.e*., integrin signalosome) is complex and cell-specific, but usually involves autophosphorylation of focal adhesion kinase (FAK) and subsequent recruitment and activation of Src family kinases (SFK) [24].

## ITGB1 and cell adhesion, spreading, and blood vessel wall stability

**Fig. 2:**
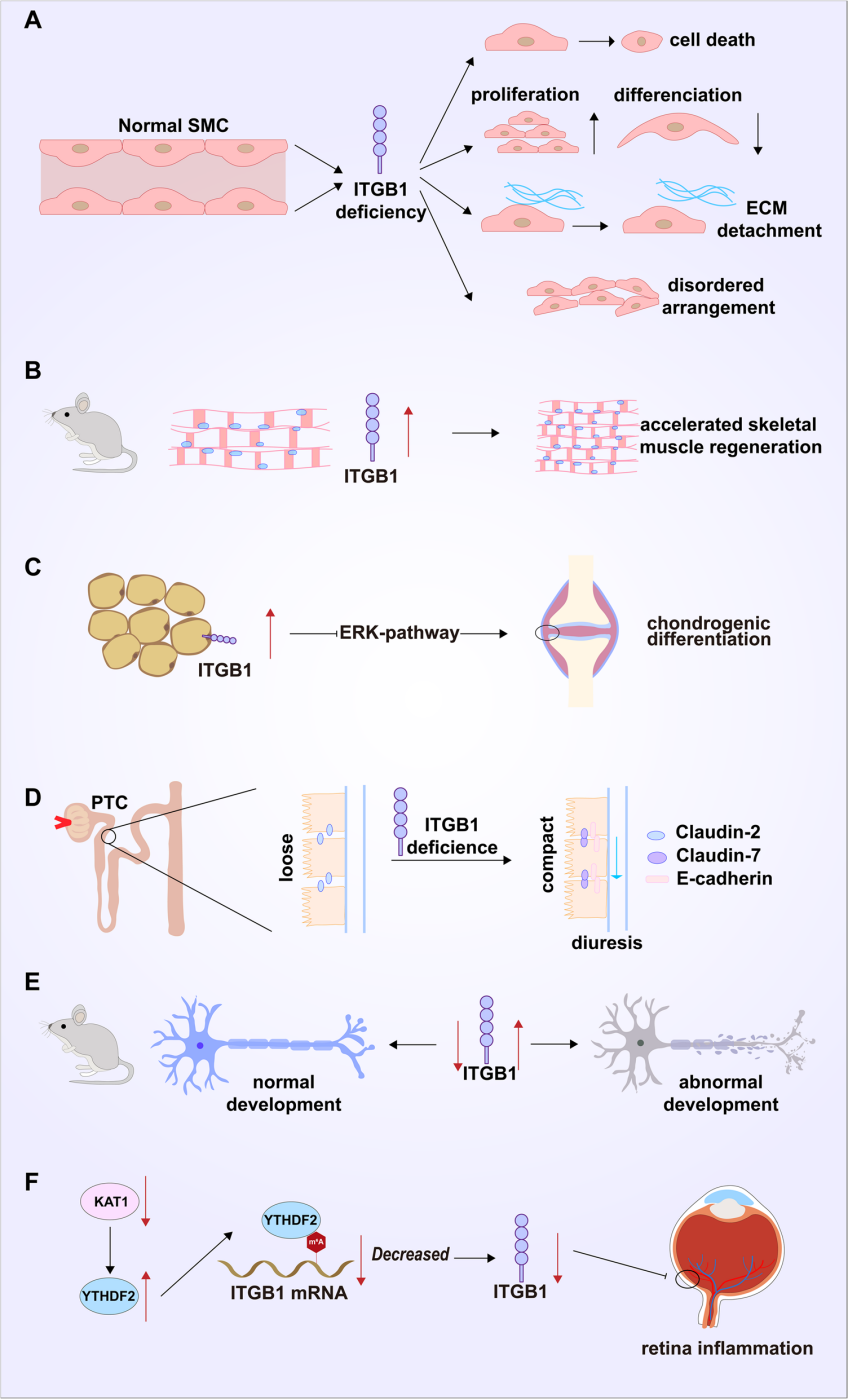
ITGB1 and physiological function, benign disease. **A** ITGB1-deficient VSMCs have an enhanced cell proliferation capacity, while the ability to differentiate and support blood vessels is impaired. **B** Upregulation of the ITGB1 expression accelerates skeletal muscle regeneration in mice. **C** Upregulation of the ITGB1 expression in adipose derived stem cells (ADSCs) promotes cartilage differentiation of ADSCs. **D** The deletion of the ITGB1 subunit in proximal tubular cells leads to their conversion from loose epithelial cells characterized by a low expression of E-calmodulin and claudin-7 and a high expression of claudiin-2 to very dense epithelial cells. This leads to picomolecular diuresis under basal conditions and impaired urine concentration after water restriction. **E** Inhibition of the ITGB1 expression in glial cells may lead to neurodegenerative and behavioral abnormalities in adult mice. **F** YTHDF2 induces instability of ITGB1 mRNA and leads to mRNA degradation in the form of m^6^A. After ITGB1 silencing, it inhibits the FAK/PI3K/AKT signaling pathway. Moreover, the change in the YTHDF2 activity is caused by KAT1. YTHDF2-mediated instability of ITGB1 mRNA can slow the progression of diabetes retinopathy

Vascular smooth muscle cells (VSMCs) deficient in ITGB1 lead to mural cell defects and postnatal death in mice. ITGB1-deficient VSMCs demonstrated several features of the synthetic phenotype: cell proliferation was enhanced, whereas differentiation and their ability to support the vasculature were impaired. This suggests that ITGB1-mediated cell–matrix adhesion is a major determinant of the mural cell phenotype [25]. Mutant embryoid bodies (EBs) were found to be defective in vascular development and, in addition, lower endothelial cell numbers in ITGB1-deficient EBs were attributed to increased rates of apoptosis and proliferation. Enhanced apoptosis and proliferation in ITGB1-deficient endothelial cells were associated with elevated p-eNOS and p-AKT signaling molecules, respectively [26]. For growth and survival, adherent cells need adequate integrin-mediated ECM engagement; normal cells that lack proper ECM contact anoikis. In addition to affecting the development and differentiation of VSMCs themselves, ITGB1 can also affect the adhesion of vascular cells to the ECM. The results of co-culture between endothelial cells (ECs) and VSMCs showed that upregulation of the ITGB1 expression, which enhances cell adhesion, also promoted adhesion complex assembly and stress-fiber formation in VSMCs by ECs. The PI3K/AKT pathway was more significantly activated upon VSMC adhesion [27]. The IB kinase (IKK) complex plays a significant role in the development of autophagy in mammary epithelial cells (MECs) devoid of ECM interaction. A decline in ITGA3-ITGB1 function was the primary cause of both the activation of IKK and the induction of autophagy. In isolated cells, decreased ITGA3-ITGB1 function induces autophagy by activating the MAP3K7-IKK pathway [28]. Besides, previous study indicated that in the collective cellular processes of neuroepithelial cells, placental trophoblasts and endothelial cells, the fibronectin-bound ITGB1 and ITGB3 do not act synergistically but antagonize each other: ITGB1/ITGB3 mutual antagonism controls the RhoA activity in a kindlin-2-dependent manner, balancing cell spreading, contractility and intercellular adhesion. They suggest that integrin/integrin antagonism is a universal mechanism for achieving social cell interactions important for tissue morphogenesis, endothelial barrier function, trophoblast invasion, and budding angiogenesis [29]. Meanwhile, ITGB1 is a critical sensor of force direction since it is activated by unidirectional rather than bidirectional shear stresses. Surface labelling and EC-specific gene deletion investigations in the mouse aorta implied that, while ITGB1 is not activated at sites of bidirectional flow, it is crucial for EC alignment in areas of unidirectional flow. Therefore, a crucial method for decoding flow mechanics to support vascular homeostasis involves ITGB1 sensing of unidirectional forces [30]. Skeletal muscle regeneration was accelerated in mice with upregulated ITGB1 expression, enhanced ITGB1 binding activity to laminin, and induced activation of the FAK-ERK and FAK-AKT signaling axes during myogenesis (Fig. 2) [31].

## ITGB1 and benign diseases

Prolonged disruption of the ITGB1 expression can lead to a range of disorders. The expression of ITGB1 was upregulated in adipose-derived stem cells (ADSCs) and promoted chondrogenic differentiation of ADSCs through inhibition of the ERK signaling pathway [32]. These findings are expected to guide the treatment of ADSC-based chondrogenic defects [32].

Deletion of ITGB1 subunits in renal proximal tubular cells caused them to undergo a transformation from loose epithelial cells — characterized by low expression of E-cadherin and claudin-7 and high expression of claudin-2 — to very compact epithelial cells [33]. This results in picomolar diuresis under basal conditions and impaired urine concentration following water restriction [33]. The unique mechanism by which ITGB1 controls proximal tubular cell terminal differentiation in vitro — a process independent of ITGB1 heterodimerization or cell surface membrane localization — indicates that the ITGB1 expression is essential for the terminal differentiation of renal tubular polarized epithelial cells [33]. Meanwhile, deletion of MarvelD1 in glial cells induced abnormalities in glial fibers and led to abnormal radial migration of neurons. MarvelD1 in glial cells inhibited the ITGB1 expression and phosphorylation of FAK Tyr397, which may lead to neurodegeneration and behavioral abnormalities in adult mice [34]. The clinical progression of intracerebral hemorrhage (ICH) is also associated with the expression of ITGB1 [35]; ITGB1 was also significantly downregulated in the retinal tissues of mice with diabetic retinopathy (DR) model [36]. Upregulation of ITGB1 reduced the activity and inflammatory response of retinal Müller cells (rMCs), as well as the proliferation and metastatic potential of retinal microvascular endothelial cells (RMECs) [36]. It also prevented inflammation, neovascularization, and vascular leakage in mouse retinal tissue. ITGB1 mRNA instability was induced by YTHDF2 and resulted in mRNA degradation in the form of m^6^A. ITGB1 silencing was followed by inhibition of the FAK/PI3K/AKT signaling pathway. In contrast, changes in the YTHDF2 activity are caused by KAT1. YTHDF2-mediated instability of ITGB1 mRNA can slow the progression of DR (Fig. 2) [36].

## ITGB1 and cancer metastasis

Cancer metastasis is a complex multi-step process that requires cancer cells to invade from their primary tumor site, survive in the circulation, and eventually colonize on nearby or distant organs [37]: ITGB1 is involved in each step of this process [38]. ITGB1 plays an essential role in metastasis as well, but there are variations therein, implying that the background to cancer may be relevant to the downstream pathways targeted by ITGB1.

### ITGB1 and signal transduction in cancer cells

To activate downstream pathways related to integrins, ITGB1 is controlled by several upstream genes in cancer cells. As a multifunctional protein, ITGB1 binds to many other molecules to perform different functions and is also regulated by many different transcription factors (Tables 1 and 2). In gastric cancer (GC), SERPINB5 upregulates ITGB1 and promotes epithelial mesenchymal transition (EMT) [39], while MFAP2 also upregulates ITGB1 [40]. PCDHB9 upregulates ITGB1 via p65 and stimulates the NF-κB signaling pathway [41]. The hTERT/MDM2 complex increases the ubiquitinated degradation of FOXO3a, which lessens its ability to inhibit ITGB1 transcription [42]. Additionally, m^6^A modifies the regulation of ITGB1 by decreasing RNA methylation of ITGB1 via FTO, maintaining RNA stability, and increasing ITGB1 protein levels [43].

**Table 1:** ITGB1 and its binding proteins

Interaction protein	Interaction region	Function	Ref
DCBLD2		Promotes EMT and angiogenesis	[44]
Caveolin-1		Promotes EMT and invasion	[45]
c-Met	Extracellular domains	Increases ligand-independent phosphorylation of c-Met	[46]
Osteopontin (OPN)		CAF recruitment	[47]
Periostin (POSTN)		Promotes the PI3K/AKT and Src pathways	[48, 49]
TIMP1		Activation of the PI3-K signaling pathway	[50]

**Table 2:** ITGB1 and its binding TF

Cancer type	TF name	Interaction region	Function	Ref
Pancreatic ductal adenocarcinoma (PDAC)	TF			
Gastric carcinoma (GC)	ZEB1	(− 1166 to − 1074) and (+ 9913 to + 10,010)	Promotes transcription	[51]
Hepatocellular carcinoma (HCC)	FOXO3a		Inhibits transcription	[39]
	TFEB		Inhibits transcription	[52]
Colorectal carcinoma (CRC)	YY1		Promotes transcription	[53]
Breast cancer (BC)	SRF		Promotes transcription	[54]
	FOXM1		Promotes transcription	[55]
	HIF-1α		Promotes transcription	[56]
	EZH2		Promotes transcription	[57]
Lung cancer (LC)	RBP2		Promotes transcription	[58]
Oral squamous cell carcinoma (OSCC)	ZNF750		Inhibits transcription	[59]
Ovarian cancer (OC)	ZNF304	-3392 to -3297	Promotes transcription	[60]

In hepatocellular carcinoma (HCC), IER2 [61], PECAM-1 (CD31) can upregulate the expression of ITGB1 [62], while CSN5 can downregulate ITGB1 to promote apoptosis [52], THBS4 can interact with ITGB1 to enhance activation of downstream pathways [63], and circ-PABPC1 can directly mediate the binding of ITGB1 to the proteasome to promote ITGB1 degradation [53]. In HCC cells with a hepatitis B background, HBV X protein (HBx) inhibits the TFEB expression, which in turn prevents ITGB1 degradation and activates downstream pathways [44]. In colorectal cancer (CRC), PCDHB9 decreased the ITGB1 expression but had no effect on invasive metastasis or cell proliferation. However, SDC1 prevents the ITGB1 expression and the activation of downstream pathways [64]. YY1 controls ITGB1 at the transcriptional level [65]. DCBLD2 interacts with ITGB1 and promotes EMT in drug-resistant cells [51, 65]. In pancreatic cancer, HLA-B regulates ITGB1 in opposing ways in various cell lines [54]. By upregulating ZEB1, a gradual rise in the ITGB1 expression from acinar-to-ductal metaplasia (ADM) to pancreatic ductal adenocarcinoma (PDAC) occurs, ZIP4 causes it to increase [55, 56]. In breast cancer (BC), the transcription of ITGB1 is activated by CDC42 [57], FOXM1 [66], HIF1α [67], and EZH2 [68] to support the ability of BC cells to invade and spread. Intriguingly, the role of ITGB1 differed in various BC gene-editing models [68], with total blocking occurring in the PyVmT-induced mammary tumor model as opposed to ITGB1 knock-down in ErbB2 tumors that failed to stop breast carcinogenesis and only delayed it by 30 days [68]. MUC20 caused ITGB1 to be upregulated in ovarian cancer (OC) [58]. Mutations in COL11A1 increases the ITGB1 expression in cutaneous squamous cell carcinoma (CSCC) [45]. In lung cancer (LC), ZEB1 decreases nuclear HDAC4 accumulation and promotes ITGB1 transcription [69], whereas RBP2 directly controls ITGB1 transcription [70]. In prostate cancer (PC), ITGB1 controls the CAV1 expression [71], and METTL3 maintains ITGB1 levels at m^6^A levels, which contributes to the theory of ITGB1 concentration in transcriptional modifications [59]. In renal cancer (RC), TG2 lessens ITGB1 adhesion to promote renal cancer cell invasion [72]. In glioma, FRK inhibits ITGB1 transcription, slowing tumor growth [73]. In esophageal squamous cell carcinoma (ESCC), ZNF750 suppresses ITGB1 transcription while SIPA1 inhibits the ITGB1 expression [46]. HACE1 influences the ITGB1 expression in melanoma by degrading FN1 via ubiquitination [74]. ITGB1 is upregulated by ALDH3B2 in cholangiocarcinoma [75]. It is interesting to note that c-Met can replace the α5 subunit of α5β1 to create a c-Met/β1 complex, which has a significantly stronger affinity for fibronectin than integrin α5β1 [76]. Additionally, integrin-linked kinase phosphorylates c-Met, leading to the activation of receptors that are not ligand-dependent [76]. The c-Met/β1 complex was shown by crystallography to be able to maintain a high-affinity ITGB1 configuration [76]. Recent research, however, suggests that some integrins, including α3β1 and α6β4, may inhibit cancer metastasis [77]. For particular, when cultured in vitro, highly metastatic and aggressive PC cells showed a lower expression of α3β1 and did not proliferate [78]. In addition, Liu et al. recently found that bladder cancer cells had more invadopodia when either laminin-332 or α3β1 was depleted [79]. Oncological processes that are invasive are driven by the cross-activation of the c-Met/β1 complex and its strong affinity for fibronectin [76]. These results suggest a critical function for ITGB1 in cancer invasion and migration, primarily via an intracellular signaling cascade response.

### ITGB1 involvement in ECM remodeling

**Fig. 3:**
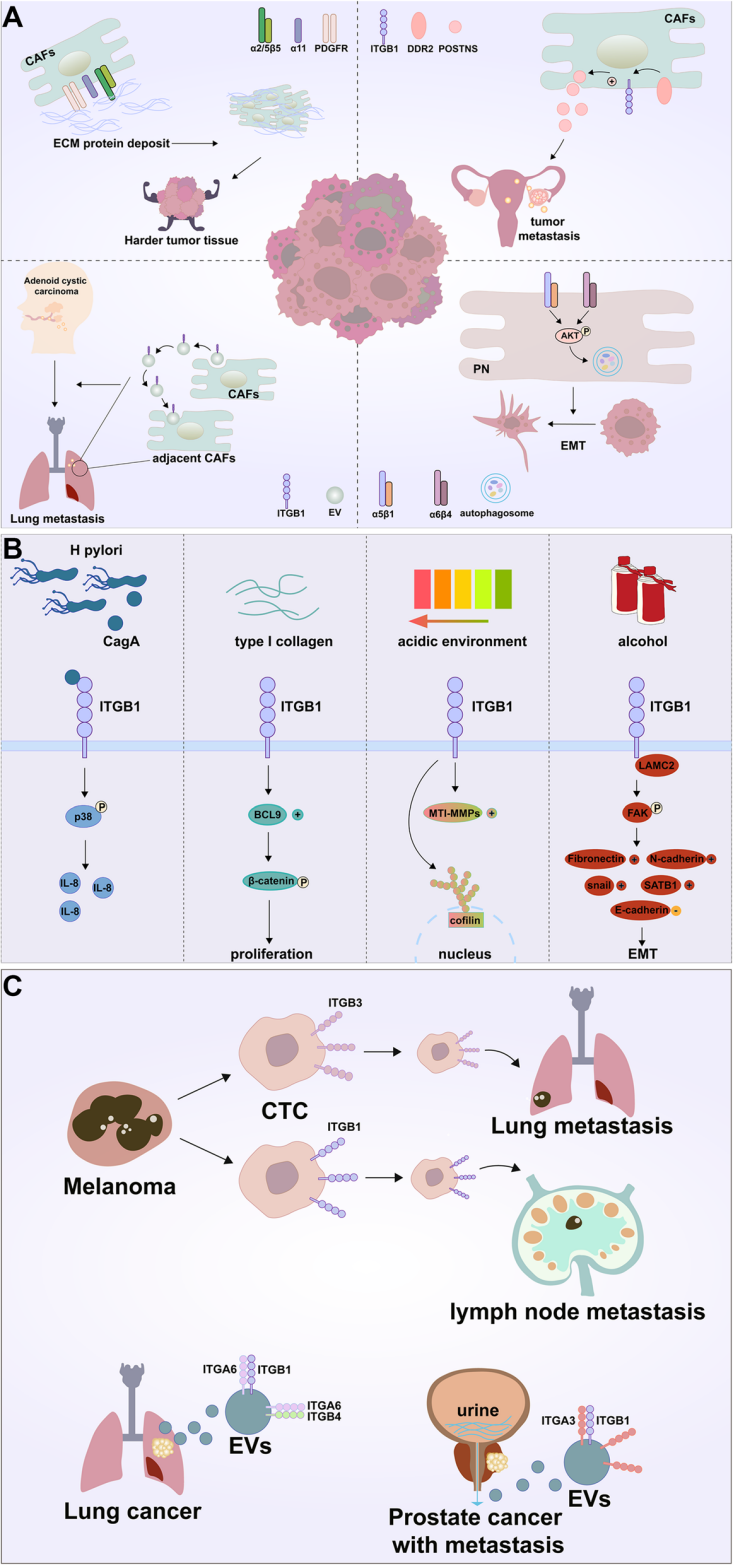
ITGB1 and cancer metastasis. **A** Tumor associated fibroblasts express ITGB1, promoting matrix remodeling and matrix deposition, thereby increasing the hardness of tumor tissue. The fibrin matrix is arranged by increasing PDGFR mediated contractility and traction, and then α5/β 1 integrin is converted into fibronectin. Meanwhile, integrins can also be expressed in CAFs to affect tumor cells. In OC, CAF increases the expression level of ITGB1 through DDR2, further upregulating the expression of POSTN in CAF, and thereby promoting the invasive metastasis of OC cells. In CRC, PN in CAFs can pass α5/β1 or α6/β4 integrin leading to AKT phosphorylation, thereby affecting autophagy mediated invasive metastasis of EMT and CRC cells. Extracellular integrins from CAFs α2/β1 integrin are absorbed by lung fibroblasts and trigger TGF- β; during signaling, the salivary gland cystic carcinoma undergoes metastasis. **B** In GC, the tumor protein CagA is introduced into host cells through *Helicobacter pylori*. The binding of CagA and ITGB1 leads to p38-mediated IL-8 production. In the presence of type I collagen, ITGB1-positive GC cells facilitate the expression of BCL9L through ITGB1, thereby activating the β-catenin signaling pathway and enhancing the ability of cells to form colonies and proliferate. In LC, the ITGB1-actin-MT1-MMPs/cofilin/F-actin signal axis promotes cancer cell movement in an acidic microenvironment. In CRC, alcohol can promote the interaction between LAMC2 and ITGB1, increasing p-FAK/FAK, snail, fibronectin, N-cadherin, and SATB1, while reducing E-cadherin. **C** The integrin produced on circulating tumor cells (CTC) significantly enhances the ability of primary tumor cells to migrate to specific organs. Melanoma cells expressing ITGB3 preferentially metastasize to the lungs, while melanoma cells expressing ITGB1 preferentially metastasize to lymph nodes. Extracellular vesicle integrin promotes the formation of pre metastatic niches by interacting with cells or ECMs in specific tissue regions. Exons produced by LC cells α6/β4 and α6/β1 integrin help colonize cancer cells that migrate in the bloodstream. Patients with prostate cancer metastasis show higher levels of ITGA3 and ITGB1 in their urine extracellular vesicles

An increasing number of studies have revealed that integrins interact with a variety of ECM elements, activate signaling molecules or pathways relevant to metastasis, and cause cancer cells to invade and migrate to nearby tissues [80]. In recent years, various studies have reported that ITGB1 is involved in ECM remodeling and that tumor microenvironment (TME) is rich in ECM components such as collagen, fibronectin, and laminin [24], thereby providing a beneficial microenvironment for tumor metastasis [81]. Cancer-associated fibroblasts, the most numerous tumor stromal cells in TME, for example, express a range of integrins, including integrins αvβ3 [82], α5β1 [83], and α11 [84, 85], which are involved in the assembly of fibronectin in the ECM and can promote the conversion of fibronectin matrix to fibronectin and the deposition of CAFs in the tumor stroma [38]. Integrins promote stromal remodeling and stromal deposition, which increases the stiffness of tumor tissue [86]. Studies indicated that CAFs align the fibrin matrix by increasing non-muscle myosin II and PDGFR-mediated contractility and traction, which are then converted to fibronectin via α5β1 integrins [83]. Integrin α6β1 is a laminin receptor found on pericytes (PNs) that regulates PDGFR and basement membrane structure and is crucial to the integrity of tumor vessels and PN recruitment [47]. It is worth noting that tumor cells recruit CAFs and promote their survival by expressing integrins [48]. Overexpression of integrin α9β1 in BC promoted the recruitment of CAFs [49]. Meanwhile, integrins can also be expressed in CAFs to affect tumor cells. In OC, CAFs increase the expression level of ITGB1 through DDR2, further increasing the expression of POSTN in CAFs, which in turn promotes the development of invasive metastasis in OC cells [87]. In CRC, PNs in CAFs can lead to phosphorylation of AKT via α5β1 or α6β4 integrins affecting autophagy-mediated EMT and invasive metastasis in CRC cells [88]. In summary, these findings showed that CAFs and cancer cells may communicate with one another due to integrin-mediated ECM remodeling in the TME, thereby supporting the progression and metastasis of cancer, and ITGB1 is involved in important processes (Fig. 3A).

In GC, gastric *H. pylori* infection is a significant risk factor for developing GC. The tumor protein CagA is introduced into host cells through the type IV secretion system (T4SS) carried by *H. pylori*. CagA303-456aa triggered p38 and ERK1/2 phosphorylation, and that ITGB1 deficiency decreased CagA303-456aa activation of p38 phosphorylation. The possible mechanism is that the binding of CagA to ITGB1 leads to p38-mediated IL-8 production, as ITGB1 acts as a receptor for CagA. Meanwhile, the activation of ERK phosphorylation by CagA303-456aa is not dependent on ITGB1 [89]. Lee et al. found that ITGB1-positive GC cells upregulated the BCL9L expression through ITGB1 in the presence of type I collagen, which in turn activates the β-catenin signaling pathway and enhances the ability of the cells to form colonies and proliferate. Subsequently the anti-apoptotic protein BCL2 is further upregulated by intranuclear β-catenin, which would afterwards result in chemotherapy resistance in GC [90]. In LC, ITGB1-actin-MT1-MMPs/cofilin/F-actin signaling axis promotes cancer cell motility in an acidic microenvironment [91]. In CRC, CD133^−^ CRC cells can restore tumorigenic potential and stem cell-like characteristics [92]. The three-dimensional (3-d) ECM can mediate cytoskeletal F-actin bundling via biomechanical pressures linked with the receptor ITGB1, causing the cytoskeleton to release the E3 ligase TRIM11 and degrade the glycolytic rate-limiting enzyme phosphatidylinositol (PFK) [92]. As a result, PFK inhibition increased glycolysis and upregulated HIF1α, promoting the reprogramming of stem cell transcription factors and aided tumor growth in patients [92]. In addition, alcohol encourages the interaction between LAMC2 and ITGB1, increasing p-FAK/FAK, snail, fibronectin, N-cadherin, and SATB1 while decreasing E-cadherin in the alcohol group compared to the non-alcohol group [93]. It suggests that alcohol may encourage CRC metastasis by molecular processes that change the pre-metastatic environment [93]. In PDAC, activation of pancreatic stellate cells (PSCs) is a feature. GAL3 was found in PDAC, and PSC proliferation and invasion were increased when PDAC cells expressing different levels of GAL3 were cultured alongside PSCs. Through ITGB1, GAL3 induces the transcription of IL-8 on PSCs, which in turn activates NF-κB through ILK. GAL3 inhibitors slow the development and metastasis of orthotopic tumors formed in mice by the co-implantation of PDAC and PSC cells [94]. In PC, Aberg et al. discovered that TF/FVIIa-induced activation of ITGB1is necessary for the transactivation of IGF-1R [48]. Through its scaffolding structural domain, the CAV1 protein suppressed IGF-1R activation in dormant cells. By activating SFK and phosphorylating CAV1 on tyrosine 14, TF/FVIIa/ITGB1 overcame this inhibition [48]. Therefore, the downregulation of ITGB1 or the overexpression of CAV1 can prevent the anti-apoptotic effects of FVIIa or the production of CCND1-mediated by IGF-1R [48]. In BC, the fascial system increased the expression of ITGB1 in BC cells and enhanced their adhesion to different ECM matrices. Furthermore, the expression of fascial protein-mediated ITGB1 promoted breast self-renewal and chemoresistance. Fascia was significantly associated with the ITGB1 expression, and their co-expression was significantly associated with shorter disease-free and overall survival rate [49]. Lung metastasis and the migration of glioblastoma and osteosarcoma cells were also facilitated by the association between α9β1 integrin and TN-C [87].

Additionally, it has been noted that the ability of primary tumor cells to metastasize to specific organs was significantly enhanced by the integrins produced on circulating tumor cells (CTC) [95]. For particular, the kind of integrins produced by circulating melanoma tumor cells determines how likely melanoma is to metastasize to various organs [96]. Melanoma cells express ITGB3 preferentially metastatic to the lungs, whereas melanoma cells expressing ITGB1 preferentially metastasize to lymph nodes [97, 98]. CTC invasion can also be facilitated by the integrin of target organ endothelial cells (control of the microvasculature may be the underlying mechanism thereof [96]). As a result of their interaction with specific ECM elements in the tissue milieu, integrins facilitate the development of pre-metastatic niches, which in turn creates an ideal “soil” for cancer cells to spread to and colonize particular organs (Fig. 3B).

### ITGB1 in cancer-derived exosomes aid metastasis

According to recent research, extracellular vesicle integrins, particularly exosomes, promote the formation of pre-metastatic niches by interacting with cells or ECM at particular tissue regions [37, 99]. Cancer cells secrete exosomes, which are tiny membrane vesicles (30–100 nm in diameter) [100]. Exosomes are both short and long-distance intercellular communication mediators by encapsulating functional biomolecules (such as proteins, lipids, RNA, and DNA). They do this by altering the ligand-receptor interaction and/or cargo release in the receiving cells [100]. Due to their capacity to modify the ECM, integrins — which are the most commonly expressed receptors on the surface of exosomes — have been shown to play a significant role in the metastasis of exosomes [101]. The first determinants of organotypic tumor spread to be discovered by Hoshino et al. are exosomal integrins secreted by tumor cells [37]. Exosomes α6β4 and α6β1 integrins produced by LC cells, which assist in the colonization of cancer cells that have migrated throughout the blood stream, are preferentially absorbed by S100A4 fibroblasts and SPC epithelial cells [37]. Cancer exosomes upregulate fibroblast proteins implicated in focal adhesion (α2/α6/αv, β1/β4/β5, EGFR, CRK), regulators of actin cytoskeleton (RAC1, ARF1, ARPC3, CYFIP1, NCKAP1, ICAM1, and ERM complex), and signaling pathways (MAPK, Rap1, RAC1, and Ras) important in pro-invasive remodeling of ECM, which leads to the creation of pre-metastatic niches [102]. In PC, Bijnsdorp et al. found higher levels of ITGA3 and ITGB1 in urinary exosomes of metastatic patients compared to benign prostate hyperplasia (BPH) or PC. Inhibition of ITGA3 or ITGB1 in exosomes prevented the migration and invasion of non-cancerous prostate epithelial cells (prEC) [103]. In BC, Tan et al. discovered that ITGB1 from breast cancer cells could be transmitted to recipient cells via sEVs and further increase MCF7 cell migration, while inadequate glycosylation was found to be present during the BC growth. In addition to showing that ITGB1 is a target protein for bisecting GlcNAc, significant bisecting GlcNAc structures and decreased amounts of its glycosyltransferase MGAT3 were found in breast cancer cell lines, tissues, and serum. Bisecting GlcNAc may prevent ITGB1 from forming branching GlcNAc structures in donor cells via MGAT3, and in recipient cells, ITGB1 might prevent sEVs from acting as pro-metastatic sEVs by blocking their ability to attach to Galectin-3 [104]. Notably, pre-metastatic niche development in the lung can be induced by exosomes from CAFs. Adenoid cystic carcinoma (ACC) develops metastases when exosomal integrin α2β1 from CAFs is taken up by lung fibroblasts and triggers the TGF-β signaling pathway (Fig. 3A) [105].

The interaction between integrins and ECM has been shown to enhance the intracellular movement and plasma membrane production of integrins via the endosomal pathway in addition to activating metastasis-related signaling pathways [88]. The endosomal pathway can create integrins that can control the development and modification of proteins in the ECM, enabling the invasion of surrounding tissues by tumor cells [88]. Rab-coupling protein (RCP) and diacylglycerol kinase-dependent endosomal pathway recycling were indicators of the increased invasiveness of mutant p53 tumor cells [88]. RCP has a reputation for being able to regulate integrin recycling [89]. Through the activation of RCP-dependent integrin recycling, mutant p53 tumor cells promoted invasiveness and migratory function by producing exosomes that were horizontally transmitted to other tumor cells [88]. In 3-d fibronectin-rich ECM, RCP-driven endocytic recycling of α5β1 integrins facilitated ARP2/3 complex-independent OC cell motility [90]. Further investigation proved that the α5β1 integrin pathway was a critical channel for cancer cells to promote invasive migration, and ROCK-dependent phosphorylation and FHOD3-dependent activation were the important mechanisms underpinning such behavior [90]. These data imply that integrins, primarily through their interactions with ECM, play significant roles in cancer migration and invasion (Fig. 3C).

In conclusion, tumor integrins are important molecules that regulate the spread of tumor cells to particular organs.

## ITGB1 and cell proliferation and apoptosis

**Fig. 4:**
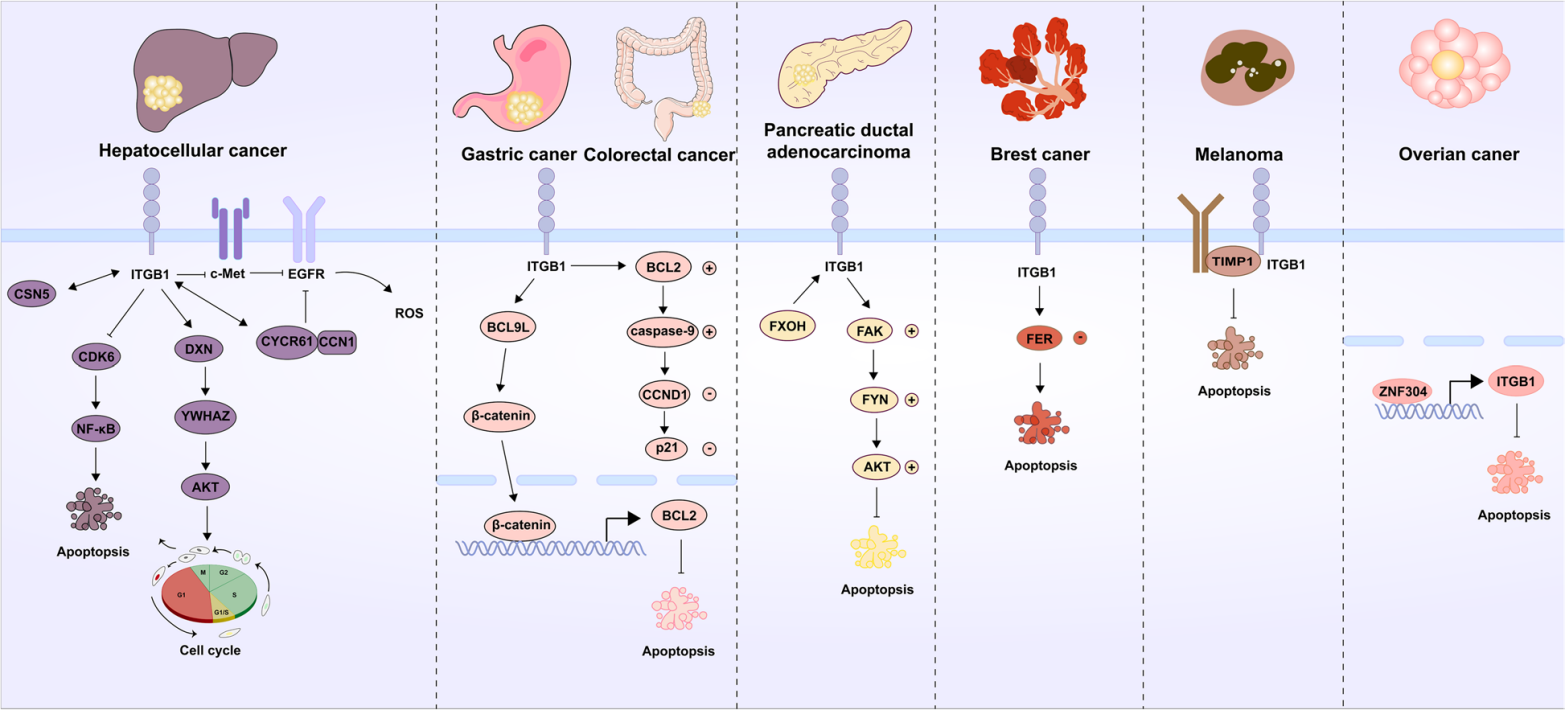
ITGB1 and cell proliferation and apoptosis. In cancer cells, ITGB1 is involved in controlling cell proliferation or apoptosis pathways. The downregulation of ITGB1 in liver cells leads to dephosphorylation of c-Met and EGFR, thereby inhibiting cell proliferation. The ITGB1/PXN/YWHAZ/AKT axis promotes the development of HCC by accelerating the cell cycle process. In addition, knocking down CSN5 can lower ITGB1 and CDK6, activating the NF-κB and other signaling pathways that lead to cell apoptosis. CYR61/CCN1 can be achieved by combining α1/β1 integrin, inhibition of the ITGB1 expression, and stimulation of intracellular ROS accumulation can suppress the activation of the EGFR signaling pathway and the proliferation of HCC cells. In GC, BCL9L is activated by ITGB1, β-catenin signaling pathway can enhance cell cloning and proliferation, and then the anti-apoptotic protein BCL2 is also activated. In CRC, overexpression of ITGB1 increases the BCL2 level while reducing the expression levels of autophagy related proteins such as Bax, caspase-3, and caspase-9. In addition, it increases CCND1 while reducing the level of p21. In PDAC, FxOH reduces apoptosis of PANC-1 cells by upregulating the expression of ITGB1, FAK, PXN, FYN, AKT, and PPAR. In BC, increased expression of α6/β1 integrin can decrease the number of non-receptor tyrosine kinase FERs in the cytoplasm, thereby weakening the ability of cells to resist apoptosis. Melanoma cells produce TIMP1, which helps them overcome apoptosis by constructing complexes with CD63 and ITGB1. In OC, ZNF304 can enhance cellular resistance to apoptosis by controlling ITGB1 transcription

In both normal tissue cells and cancer cells, ITGB1 participates in the pathways that control cell proliferation or apoptosis [106]: in normal cells, multiple receptors including EGFR, c-Met, PDGFR, and VEGFR directly interact with integrins to activate the cell proliferative activity, and do not involve their normal ligands. Liver regeneration experiments showed that downregulation of ITGB1 in hepatocytes led to dephosphorylation of c-Met and EGFR, thereby suppressing cell proliferation [107, 108]. IKK complex plays a significant role in the development of autophagy in MECs devoid of ECM interaction. A decline in α3β1 integrins function was the primary cause of both the activation of IKK and the induction of autophagy. In isolated cells, decreased α3β1 integrin function induces autophagy via activating the MAP3K7-IKK pathway [28]. Turning attention to the tumor, in HCC, ITGB1/PXN/YWHAZ/ AKT axis promotes HCC advancement by speeding up the cell cycle process [109]. Furthermore, CSN5 knockdown downregulates ITGB1 and CDK6. It causes the activation of NF-κB and other signaling pathways leading to apoptosis [52]. CYR61/CCN1 can inhibit the activation of the EGFR signaling pathway and the proliferation of HCC cells by interacting with the α1β1 integrins, suppressing the expression of ITGB1 and stimulating the accumulation of intracellular ROS [110, 111]. In GC, as mentioned above, BCL9L that is activated β-catenin signaling pathway can enhance cell cloning and proliferation, and then the anti-apoptotic protein BCL2 that is also activated β-catenin can be upregulated [90]. In CRC, overexpression of ITGB1 in HT29 cells increased the BCL2 levels, while decreasing the levels of expression of autophagy-related proteins such as Bax, caspase-3, and caspase-9. In addition, it increased CCND1 while decreasing the levels of p21. suggesting that ITGB1 may be key to controlling the cell cycle and apoptosis in CRC cells [112]. In PDAC, FxOH decreases apoptosis of PANC-1 cells by upregulating the expression of ITGB1, FAK, PXN, FYN, AKT, and PPAR, while methylselenic acid can promote entosis, a type of cell death that eliminates itself by invading surrounding cells through the internalization process, via cell detachment through downregulation of CDC42 and ITGB1 [113, 114]. In BC, increased expression of α6β1 integrins can reduce the amount of non-receptor tyrosine kinase FER in the cytoplasm, which reduces the capacity of the cell to fend off apoptosis [50]. LMTK3 interacts with GRB2 directly and then activates RAS and CDC42. The expression of ITGB1 and ITGA5 is increased as a result of LMTK3 controlling cell motility and proliferation through the CDC42-SRF route and GRB2 in the RAS-ERK/MAPK pathway, respectively [57]. Melanoma cells have been shown to produce TIMP1, which helps them overcome apoptosis by building complexes with CD63 and ITGB1 [60]. In OC, ZNF304 can enhance cell apoptosis resistance by controlling ITGB1 transcription [115, 116]. Vacuolar-ATPase inhibitors have been demonstrated to control the anti-apoptotic capacity of several tumor cells by lowering the ITGB1 activity [116]. Here, the ITGB1 protein expression and clinical characteristics in multiple cancers were summarized. Its functions and mechanisms in various types of cancers are summarized in Table 3. To provide a more succinct description, we summarized the associations between the ITGB1 expression and the clinical characteristics of various tumors in Table 4. In general, these results suggest that ITGB1 can play a significant role in the biological process of tumor proliferation or apoptosis (Fig. 4).

**Table 3:** Oncogenic/tumor-suppressive role of ITGB1 in various cancers

Cancer type	Oncogenic/tumor-suppressive	Evidence	Mechanism/pathway	Ref
Gastric carcinoma (GC)	Oncogenic	Protein expression	The FAK pathway	[36–40]
	Oncogenic	Protein expression	The BCL9L/β-catenin/BCL2 signaling pathway	[88]
Hepatocellular carcinoma (HCC)	Oncogenic	Protein expression	FAK/PI3K/AKT pathways	[42, 61, 62]
	Oncogenic	Protein expression		[41, 43, 52]
	Oncogenic	Protein expression	PXN/YWHAZ/AKT pathways	[108]
Colorectal carcinoma (CRC)	Oncogenic	Protein expression	The FAK/Wnt pathways	[63]
			EMT	[44]
				[53, 111]
			The AKT‐dependent pathway	[49]
			The F‐actin/TRIM11/PFK/HIF1α pathways	[90]
Pancreatic ductal adenocarcinoma (PDAC)	Oncogenic	Protein expression		[51, 64, 65, 117]
Breast cancer (BC)	Oncogenic	Protein expression		[54, 56, 57]
			FAK/SRC pathways	[55]
	Oncogenic	Protein expression		[47]
			RING1/Rad51 pathways	[118]
Epithelial ovarian cancer (EOC)	Oncogenic	Protein expression		[60, 66]
	Oncogenic	Protein expression	Periostin signaling in CAFs	[48]
Cutaneous squamous cell carcinomas	Oncogenic	Protein expression		[67]
Lung cancer	Oncogenic	Protein expression		[58, 97]
Prostate cancer	Oncogenic	Protein expression		[69, 70]
	Oncogenic	Protein expression	TGFβ signaling pathways	[45]
	Oncogenic	Increased activation in CAFs	High traction forces produced by CAFs were transduced to Fn pathways	[80]
Glioma	Oncogenic	Protein expression	FAK signaling pathways	[71]
Oral squamous cell carcinoma (OSCC)	Oncogenic	Protein expression		[59]
Melanoma	Oncogenic	Protein expression		[72] [50, 95]
Cholangiocarcinoma (CCA)	Oncogenic	Protein expression		[73]

**Table 4:** Expression of ITGB1 in various cancers

Cancer type	mRNA/Protein	High/low expression	Percentage	Clinical characteristics	Ref
Gastric carcinoma (GC)	Pro	High	82.50% (*n* = 160)	Lauren’s classification, differentiation degree, TNM stage	[36]
	Pro	High		Positively correlated with advanced tumor node metastases (TNM) stage and in lymphatic metastasis, but not with sex or age	[39]
	Pro	High		Elevated expression of ITGB1 in chemo-resistant (CR) tumor tissues	[88]
Hepatocellular carcinoma (HCC)	Pro	High	72.5% (*n* = 117)		[62]
Colorectal carcinoma (CRC)	Pro	High	48.7% (*n* = 66)	Significantly associated with poor prognosis	[53]
	Pro	High		Correlated with tumor recurrence	[90]
Breast cancer (BC)	Pro	High	62% (*n* = 108)	Associated with shorter overall survival	[54]
Prostate cancer (PC)	Pro	High			[45]
Melanoma	Pro	High	52% (*n* = 111)	With lymph node involvement	[96]
Lung cancer (LC)	Pro	High	38.0% (*n* = 50)		[97]

## ITGB1 and tumor stemness

Virtually all tumors in humans with cancer have subgroups of cells that are both phenotypically and functionally distinct [119]. Cancer stem cells (CSCs), a subpopulation of cells with the capacity for self-renewal and differentiation, have recently been detected. They are thought to be the primary cause of malignant features by promoting tumor development, metastasis, and medication resistance [120–122]. Cancer stemness typically increased as a tumor progress [123]. The TME, akin to regular stem cells (SCs) that are often linked to a specific local niche, controls the fate of CSCs by delivering cues to guide their biological behavior. Integrin is necessary for SCs to detect and respond to various cues in both healthy and sick tissues because it functions as the bridge for “outside-in” and “inside-out” signals [20]. Integrins have been shown to be essential for the development, progression, and differentiation of cancer, confirming their role in the development of CSC characteristics in a variety of human malignancies [124]. Research has found that integrins may act as phenotypic markers and functional regulators of CSCs, adding to the complexity of CSC regulation and providing opportunities to develop integrin-targeted therapies to prevent cancer stemness [125, 126].

Complete suppression of carcinogenesis was seen in mice that lack ITGB1 activity, in the mammary gland, tissue-specific loss of ITGB1 function can suppress the development and proliferation of CD24^hi^CD29^lo^CD61^hi^ cancer cells [127, 128]. Researchers identified CD24^+^CD44^+^ stem cells from the PANC-1 cell line and showed that these cells had a higher capacity for invasion than CD24^−^CD44^−^ cells in pancreatic cancer. The increase in the ITGB1 expression in CD24^+^CD44^+^ stem-like cells was given as the explanation for this [129]. Mechanistically, pancreatic cancer cells can activate CAFs and boost collagen production, which further promotes the self-renewal and migration of pancreatic cancer cells and increases the frequency at which cancer cells are transformed into stem cells through the FAK activation. The influence of CAFs on clonal proliferation was significantly reduced when ITGB1/FAK signaling was inhibited in pancreatic cancer cells [130]. According to a different study, pancreatic CSCs exhibit higher levels of the enzyme aldehyde dehydrogenase (ALDH), which is linked to the ability to metastasize [117]. These ALDH^+^ CSCs have higher levels of the ITGB1-FAK expression, and additional FAK inhibition can prevent the formation of clonogenic pancreatic cancer cells both in vitro and in vivo [131]. TN-C, a ligand of ITGB1 and ITGB3, is produced by breast cancer cells to encourage CSC self-renewal and to improve their capacity to initiate metastasis [84]. In CD133^−^CRC cells, ITGB1 facilitates the reprogramming of stem cell transcription factors and the recovery of tumorigenic potential and stem cell-like characteristics [92]. The downregulation of SDC1 synergistically promoted activation of ITGB1 and FAK, increasing the invasiveness of the signal and the properties of CSCs [37]. In RC, TG2 is not only critical for cancer cell adhesion, migration, and invasiveness during RC progression and propagation, but reducing TG2 expression decreases the adhesion of ITGB1, fibronectin, type I collagen, and laminin by 60% and decreases the expression of CD44, CD73, and CD105 CSC-like markers [72].

According to these results, the ITGB1 strengthens the characteristics of CSCs and promotes tumorigenesis, self-renewal, and metastasis through signaling mechanisms including FAK.

### ITGB1 functions as CSC markers

**Fig. 5:**
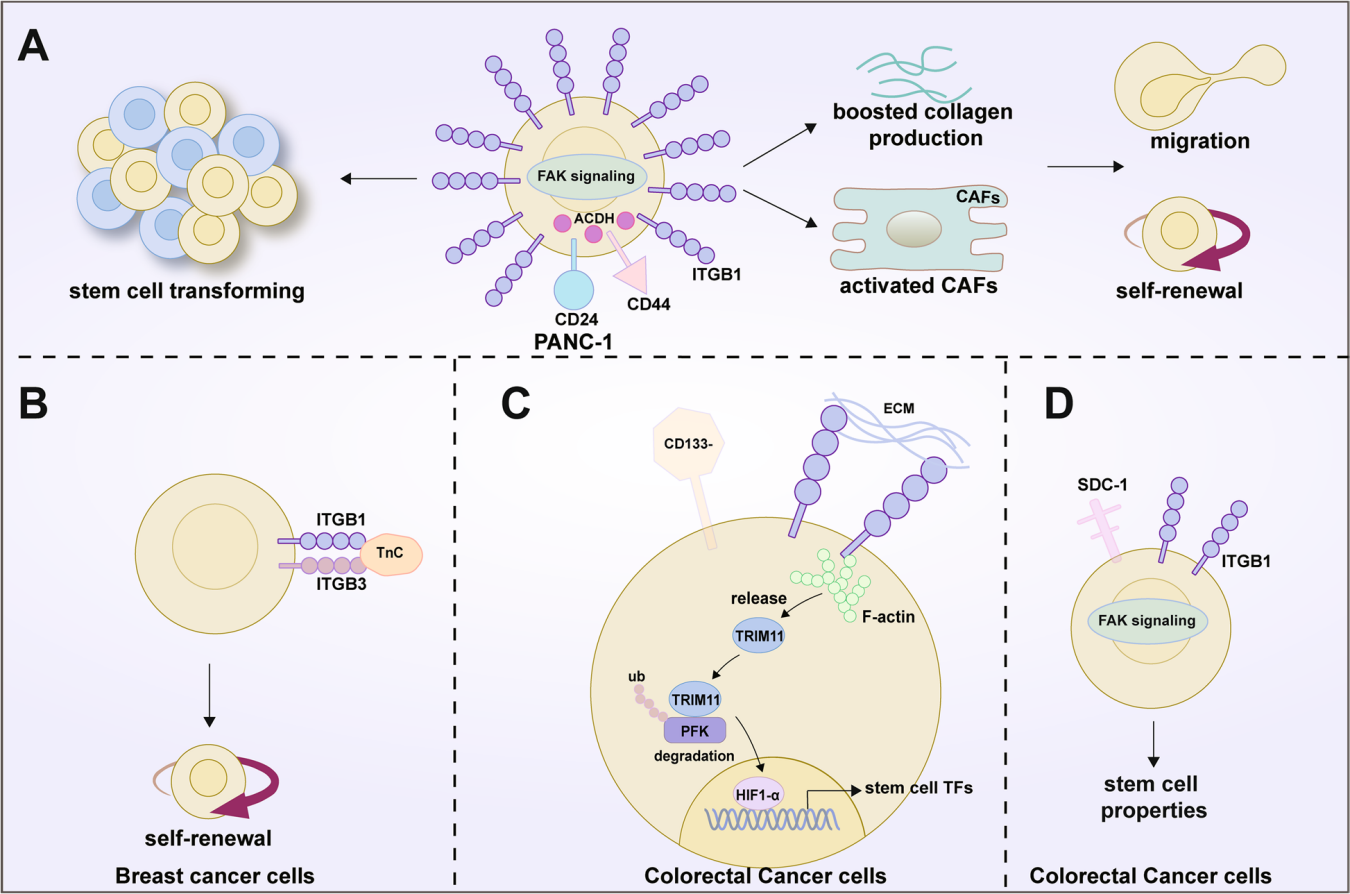
ITGB1 and tumor stemness. **A** CD24^+^CD44^+^ stem cells were identified in the PANC-1 cell line, with increased expression of ITGB1. These cells are more invasive than CD24^−^CD44^−^ cells in pancreatic cancer. These cells have the ability to activate CAF and promote the production of collagen, thus further promoting self-renewal and migration of pancreatic cancer cells, and increasing the frequency of cancer cells transforming into stem cells through FAK activation. In addition, pancreatic CSC exhibits a higher level of ALDH, which is related to its metastatic ability. Meanwhile, these ALDH^+^CSCs demonstrate high levels of ITGB1-FAK expression. **B** TN-C, a ligand of ITGB1 and ITGB3, can be produced by breast cancer cells to encourage self-renewal of CSC and enhance its ability to initiate metastasis. **C** In CD133^−^CRC cells, ITGB1 promotes reprogramming of stem cell transcription factors and restores tumorigenic potential and stem-cell-like characteristics. **D** In CRC, downregulation of SDC1 synergistically promotes the activation of ITGB1 and FAK, increasing the invasiveness of cancer cells and the advantages conferred by the use of CSCs

Several cell surface markers, including CD24, CD44, Nanog, CD90, CD133, SOX2, SOX9, ESA, and KLF4, have been successfully used in the past to identify CSC subpopulations phenotypically in cell culture and clinical samples from a variety of cancer types [123, 132–134]; unfortunately, due to their lack of organ specificity, these markers frequently also identify normal SCs, necessitating the hunt for additional markers the more thoroughly and properly to define CSCs. Owing to their crucial positioning on the cell surface and significant role in the evolution of tumors, integrins are another category of transmembrane proteins that have garnered considerable interest in previous studies [135]. Nevertheless, it is mainly other members of the integrin family that can be used as surface markers, such as ITGA6 [136], ITGA7 [137], and use of ITGB1 has not been reported: available studies suggest that its function is focused on regulating the development and progression of tumor CSC. Typically, CSCs co-opt niche-integrin signals to support their growth [20]. Squamous cell carcinoma (SCC) has two highly tumorigenic subgroups that can be identified by high or low CD34 expression but only a high integrin content [138]. Regardless of the degree of CD34 expression, only the α6^hi^β1^hi^ cell-initiated tumors. This implies that high levels of b1 integrins are a more appropriate marker of CSCs that cause tumors in SCCs. [138]. It is fascinating to note how differently the reciprocal states of α6^hi^β1^hi^ CD34^hi^ and α6^hi^β1^hi^ CD34^low^ are regulated at a molecular level. By binding to its ligand FN1, activated ITGB1 in one state can result in certain CD34^hi^ and CD34^low^ CSC populations, but in the other, active TGF/TRII signaling selectively shadows α6^hi^β1^hi^ CD34^hi^ cells, reducing their stemness and driving differentiation. Undoubtedly, FAK signaling is key to the tumorigenic characteristics of both CSC subpopulations (Fig. 5) [138].

Overall, these data show that ITGB1 is involved in nearly every stage of cancer progression, from the formation of primary tumors to metastasis [24]. Notably, several integrin-derived signals are regularly reported as being crucial for CSC function, which is consistent with the hypothesis that tumor stemness is a key driver of cancer evolution. As a result, targeting specific integrins and inhibiting CSCs by modifying cell adhesion and integrin/ECM interactions is another possible therapeutic approach [20, 24, 139]. However, studies have also shown that integrins can control tumor stemness without interacting with the ECM, and that this adhesion-independent activity activates pathways that are distinct from common signaling cascades and cytoskeletal connections [20]: this presents a considerable obstacle to the creation of therapeutic medicines that target the ligand or receptor of ITGB1.

## ITGB1 and cancer drug resistance

Many studies have found that drug resistance in cancer cells is controlled not only by internal factors (gene mutations, copy number variations, and epigenetic modifications, for example), but also by external factors (tumor microenvironment, ECM, etc.) [135]. Drug resistance can develop in cancer cells in a variety of ways, including adaptation to environmental signals, the reactivation of relevant pro-survival signals and anti-apoptotic programs, the preferential selection of drug-resistant subgroups, and induced changes in the microenvironment [118, 135]. The different mechanisms underlying ITGB1-mediated primary and adaptive drug resistance are associated with a particular tumor type or the mode of action of a given medication.

### Abnormal activation of intracellular ITGB1 and cancer drug resistance

The abnormal activation of ITGB1 promotes downstream signaling, enhancing the tolerance of cancer cells to radiation. The capacity of matrix-bound ITGB1 to stimulate DNA repair and pro-survival signaling causes resistance to radiotherapy in a variety of malignancies [140–142]. Additionally, aberrant cell proliferation signals are activated when ITGB1 is overexpressed and downstream signaling pathways are activated, obviating the effects of drugs that block its function, for instance, in PDAC, ITGB1-driven Src-AKT overactivation results in EGFR ligand-independent proliferative signaling, which compromises the effectiveness of cetuximab therapy [143]. An intriguing discovery was that NRP1 physically interacts with active ITGB1, which may be prevented by NRP1 targeting peptide TPP11 [143]. To block active ITGB1-driven signaling and block EGFR signaling concurrently, they produced the dual-targeting antibody Ctx-TPP11, which could have inhibitory effects on PDAC proliferation both in vitro and in vivo. This might represent a new approach to PDAC therapy [143]. Additionally, by activating CDC42 molecules on the PI3K-p110β signaling pathway, ITGB1 enhances PDAC resistance to gemcitabine [144]. ZIP4 elevated the ZEB1 expression, which in turn stimulated the expression of ITGA3 and ITGB1. The expression of the gemcitabine transporter ENT1 was then suppressed by enhanced ITGA3/ITGB1 signaling via JNK, which reduced the quantity of drug uptake by the cells [56]. Targeted suppression of ITGB1 increased the susceptibility of cancer cells to chemotherapeutic drugs in head and neck cancer [142, 145]. Tumor cells grown on 3-d laminin-rich ECM cultures were radiosensitive to dual-targeted treatment with AIIB2 (targeting ITGB1)/imatinib (targeting c-Abl, a tyrosine kinase), which also significantly reduced DNA damage repair in head and neck cancer cells [145]. The simultaneous targeting of ITGB1 and EGFR also produced radio-sensitizing effects on head and neck cancer, aIIB2 increased cytotoxicity and radio-sensitization in a variety of head and neck cancer cells when combined with cetuximab and X-rays [118, 146]. In fact, it has been suggested that ITGB1 may enhance resistance to antiangiogenic therapy by elevating a number of malignant programs made possible by interactions with the TME [147]. The development of vasculogenic mimicry (VM) by cancer cells is one program, and ITGB1 is a crucial regulator thereof [148]. In HCC, the α5β1 integrin dimer plays a pro-angiogenic role that enables cancer cells to produce angiogenic mimics and display angiogenic-like characteristics when overexpressed. Compared to conventional VEGF-dependent vasculature, these vessels are unique. Cancer cells without ITGB1 are unable to construct VM networks, but when ITGB1 is added back in, these cells can do so. When it comes to cell adhesion, signaling pathways, cytoskeletal structure, and force production, the physical interactions of α5β1 integrin with their ligands are essential [149].

In GBM, For the first time, Carbonell et al. demonstrated a relationship between anti-angiogenic therapy resistance and enhanced ITGB1 expression, mobility, and turnover in focal adhesions. Inhibiting ITGB1 can be used to overcome resistance to anti-angiogenic therapies. Unlike natalizumab (which targets ITGA4 and prevents the ability of α4β4 or α4β7 integrin to bind VCAM-1), a single treatment targeting ITGB1 would prevent tumor cells from adhering to a number of ECM ligands, including fibronectin, collagen IV, and laminin [150]. Another study revealed that the α5β1 integrin, which blocks p53 signaling, is associated with temozolomide resistance [151]. Bortezomib (BTZ), is a key adhesion receptor mediating multiple myeloma (MM) cell-chromosome interactions and MM cell survival. BTZ suppresses cell adhesion-mediated drug resistance (CAM-DR) and MM cell apoptosis by downregulating the expression and function of α4β1 integrin. Not only had α4β1 integrin expression been restored in BTZ-resistant MM cells, but it had also increased to a level higher than that of the parental cells. In comparison to parental cells, the NF-κB pathway is more robustly activated in BTZ-resistant MM cells, which contributed to an increase in the expression of ITGA4 and α4β1 integrin-dependent MM cell adhesion [152]. DCBLD2, influential in 5-FU resistance in CRC, promotes angiogenesis and EMT, which, in turn, promotes the progress of CRC. ITGB1, with which DCBLD2 interacts, is a critical signaling element of the focal adhesion pathway, which is an essential path known to regulate EMT [51]. ITGB1 is frequently upregulated in OC and increases tumor cell invasion by increasing the expression of MMP-2 and MMP-9. Fludarabine inhibits the ITGB1/FAK/STAT1 signaling pathway and suggests that anticancer therapy with bevacizumab is effective in reducing tumorigenesis and disease progression [153].

It has been observed that, in T acute lymphoblastic leukemia (T-ALL), ERK activation by α2β1 integrin mediates resistance to doxorubicin [154]. Engagement of the α2β1 integrin by stromal VCAM1 and activation of CXCR4 together promote survival signaling via the Syk tyrosine kinase in chronic lymphocytic leukemia (CLL). As a result, fludarabine sensitizes CLL cells in vitro when Syk is inhibited [155]. Lapatinib and trastuzumab are more sensitive to ErbB2-positive breast cancer cells when the laminin-binding integrins (α6β4 integrin and α3β1 integrin) are inhibited in normal culture conditions [156]. Additionally, FAK and Src are activated in BC cells that have been chosen for their resistance to both medications. Depletion of ITGB1 or pharmacological inhibition of FAK prevents the growth of these drug-resistant cells in 3-d Matrigel [157].

The combination of targeted ITGB1 and other anti-tumor medicines (radiotherapy, chemotherapy, and targeted therapy), has the potential to overcome tumor resistance. Integrin is a promising anti-tumor target.

### Tumor drug resistance promoted by ITGB1-ECM cross-talk

Numerous studies have discovered that the integrin-ECM interaction is essential for the development of drug resistance in malignancies [158, 159]. Resistance to sorafenib in triple-negative breast cancer (TNBC) was mediated by activation of ITGB1 and its downstream effector JNK in the collagen-rich microenvironment. In addition, the interaction between ITGB1 and Matrigel that can activate the ABCC1 drug transporter may be the cause of Adriamycin resistance in patients with T-cell acute lymphoblastic leukemia [160]. Recent research has revealed that dexamethasone increased the levels of β1, α4, and α5 integrin in OC cells and improved the adhesion of cancer cells to ECM, causing resistance to drugs such as cisplatin and paclitaxel that lead to apoptosis in cancer cells [161]. Furthermore, research has found that long-term administration of trastuzumab + pertuzumab + buparlisib (PI3K inhibitors) in combined-use treatment results in buparlisib-resistant tumors in HER2/PIK3CA H1047R transgenic mice with BC [162, 163]. According to RNA sequencing, the integrin β1/Src signaling pathway was activated together with a considerable upregulation of the ECM and cell adhesion genes [162]. It was important to note that these drug-resistant tumor cells were sensitive to buparlisib in 2-d culture but only demonstrated resistance thereto when coated on collagen or reintroduced into mice [162]. This finding suggested that the major regulatory route mediating the HER2-positive breast cancer resistance to anti-HER2 and anti-PI3K inhibitor combo-therapy was collagen/integrin β1/Src signal transduction. Therefore, achieving homeostasis in the ECM by reducing or inhibiting ITGB1-mediated ECM stiffness and degradation would be a method exhibiting a certain potential. This will improve the penetration of anti-tumor medications.

## ITGB1 and therapy: from bench to clinic

ITGB1 is considered as a potential ideal treatment target because of its significant function in different pathological conditions and many malignancies, as well as the fact that its ligand binding and regulatory sites are extracellular (which may enable ITGB1 to access numerous therapeutic interventions) [24].

In the study of cancer-related mechanisms, Dihydroartemisinin (DHA), a predominant phytoconstituent in *Artemisia annua* L. (a plant widely used as a traditional medicine in China) was used in DHA (1–100 μM) treatment and shown to have inhibited cell proliferation in a dose-dependent manner. The results showed that DHA decreased the protein levels of FN1 and ITGB1 and interfered with the PI3K-AKT signaling pathway in HCC [164]. In CRC, Ropivacaine, an anesthetic drug, decreased the expression of ITGB1 and affected the phosphorylation of AKT/FAK/ERK, a downstream pathway of ITGB1, serving as a tumor suppressor and thereby decreasing the development and progression of CRC [165]. In endometrial cancer (EC), the EM2D9 monoclonal antibody may regulate the migration of endometrial cancer cells through a complex of CD151 and α5β1 integrin. We also found that EM2D9 regulates the FAK signaling pathway through α5β1 integrin [166]. OS2966, a neutralizing ITGB1 monoclonal antibody, attenuates aggressive tumor phenotypes in vitro and inhibits growth of antiangiogenic therapy-resistant tumor xenografts in vivo [167], constituting a potential therapeutic opportunity. Volociximab, an antibody that blocks α5β1 integrin, has been shown to reduce angiogenesis, preventing the growth of tumors in a number of xenograft models [168]. These investigations suggest that ITGB1 may be a viable therapeutic target, but this is only applicable in the laboratory. If a targeted therapy for ITGB1 is to be used in a clinical setting, much more research is required.

Targeting the integrins themselves is currently the primary therapeutic approach for medications or inhibitors. Clinical trials using this therapeutic approach, though, have faced challenges. MINT1526A (anti-α5β1 integrin monoclonal antibody) was well tolerated and exhibited a potential combination effect in a Phase-I study (NCT01139723), but it was not possible to distinguish it from bevacizumab monotherapy [169]. The aforementioned finding simply demonstrates the potential clinical utility of integrating integrin-targeted therapy with chemotherapeutic medicines; nevertheless, more useful and efficient integrin-specific targeting medications remain to be developed. By December 2020, at least 130 clinical trials of integrin-targeted medicines had been conducted, but only six integrin inhibitor medications have been released to market. Four integrins (αIIbβ3, α4β7, α4β1, and αLβ2) are targeted: three of these are antibodies and three are small molecules, while many others are mentioned in preclinical studies in academic and industrial settings. The small medicines tirofiban (Aggrastat), etibatide (integrin), and the antibody abciximab (ReoPro) were the first inhibitors of integrin aIIb3. All three medications were used to treat acute coronary syndromes, prevent thrombosis during high-risk coronary angioplasty, or treat thrombotic cardiovascular events [170].

Natalizumab (Tysabri) with vedolizumab (Entyvio) is a pan-ITGA4 inhibitory antibody that inhibits ligand binding to α4β7 integrin and α4β1 integrin. It is commonly used to treat multiple sclerosis (MS) (passing the blood–brain barrier) and Crohn’s disease (reducing T-cell homing to the gut). Despite the documented prevalence of fetal progressive multifocal white matter encephalopathy as a side effect, natalizumab has a unique efficacy in MS [171]. Unfortunately, there is a very limited success rate for medications based on current technology, whether they are antibodies or small molecules.

The development of integrin-based cancer therapies is complicated by multiple problems. First, it can be challenging to distinguish between the many (and occasionally negative) roles that integrins play in cancer and to create sensitive biomarkers that are appropriate for these roles. Second, unlike platelet and leukocyte integrins, the majority of other integrins perform redundant functions in adhesion and signaling, making it challenging to block these processes with a single drug or without compensatory upregulation of non-targeted integrins with comparable specificity and function. Last but not least, it may not be possible to simultaneously target integrin-mediated adhesion and signaling without unacceptable toxicity, especially if one wants to target the common ITGB1 or even α6β4 integrin, which has a more restricted tissue distribution but is involved in maintaining the integrity of the skin and upper gastrointestinal tract, a cross-over with normal tissue cells that jeopardizes the progress of targeted drug development, forcing researchers to consider alternative treatments [172, 173].

Unexpectedly, brand-new integrin-targeting anti-cancer medicines are beginning to take shape. Engineering nanoparticles with integrin-specific ligands to increase their affinity for cancer cells are a new therapeutic approach [174]. Integrin-targeted anticancer therapeutics are expanding in scope with the development of RNAi and nanoparticle formulations. In a xenograft model of TNBC, RGD peptide-directed siRNA-loaded nanoparticles targeting ITGB3 have significant therapeutic benefits, and lipid nanoparticles with ITGAV and ITGB1-targeted siRNA suppress hepatocellular cancer in vivo. In conclusion, integrins have significant potential as an anticancer therapy, but many unanswered questions remain. For the treatment of ITGB1, additional research is required to identify its precise ligand or to inhibit its activity through different pathways to achieve tumor suppression.

## Conclusion and perspectives

ITGB1 is a receptor protein found on cell membranes that, through its top-down activity, is essential for a variety of biological functions. Angiogenesis, neural growth, and other developmental processes are all affected when the ITGB1 expression is disrupted, which can result in the onset and progression of benign diseases as well as cancer. Its role and molecular mechanisms as an oncogenic factor have been discovered in numerous fundamental and clinical investigations, raising the possibility that it can be a potential prognostic and diagnostic marker for cancer.

Despite the efforts undertaken to investigate the biological functions of ITGB1, there remains a deal of uncertainty. While ITGB1 perception and response to ECM in TME is an important function of ITGB1, ITGB1 remains to be studied in the latest popular tumor immunological context. Most of our current information about ITGB1 comes from investigations of solid tumors. The various post-transcriptional modifications of ITGB1 and their potential functions and mechanisms warrant further investigation, as these modifications may offer new ideas for cancer therapeutic drugs targeting ITGB1. In addition, post-translational modifications are also an important factor in the stable part influencing protein expression and function. The variety of ITGB1 and its function in cancer highlight the outstanding potential of this protein as a therapeutic target. Hence, we suggest the following areas for additional study to improve the effectiveness of ITGB1-targeted medicines and comprehend the role of ITGB1 in cancer development: firstly, ITGB1-related mechanisms linking the immune and metabolic systems of cancer remain a gap that requires more research attention; secondly, knowledge derived from both in vitro and in vivo experiments remains to be translated into clinical situations. In preclinical models, various novel drugs or monoclonal antibodies have shown promising results in inhibiting cancer progression, but whether these results can be reproduced in humans remains to be tested; finally, more detailed elucidation of the mechanisms by which ITGB1 promotes cancer cell metastasis in multiple metastatic steps is required. We believe that to multiply comprehend the function of ITGB1 in disease progression will lead to the development of more creative targeted strategies and a revival in the field.

## Data Availability

Not applicable.

## Abbreviations

3D: Three-dimensional
Aa: Amino acid
ABCC1: ATP binding cassette subfamily C member 1
ADM: Acinar-to-ductal metaplasia
ADSCs: Adipose-derived stem cells
ALDH: Aldehyde dehydrogenase
ALDH3B2: Aldehyde dehydrogenase 3 family, member B2
ARF1: ADP-ribosylation factor 1
ARP2/3: Actin-related protein 2/3
ARPC3: Actin related protein 2/3 complex, subunit 3
BC: Breast cancer
BCL2: B-cell CLL/lymphoma 2
BCL9L: B-cell CLL/lymphoma 9-like
BPH: Benign prostate hyperplasia
BTZ: Bortezomib
CAFs: Cancer-associated fibroblasts
CAM-DR: Cell adhesion-mediated drug resistance
CAV1: Caveolin 1
CCND1: Cyclin D1
CDC42: Cell division cycle 42
CDK6: Cycle protein-dependent kinase 6
CLL: Chronic lymphocytic leukemia
circ-PABPC1: CircRNA-polyadenylate-binding protein 1
c-Met: Cellular-mesenchymal epithelial transition factor
COL11A1: Collagen type XI alpha 1 chain
CRC: Colorectal cancer
CRK: V-crk avian sarcoma virus CT10 oncogene homolog
CSCC: Cutaneous squamous cell carcinoma
CSCs: Cancer stem cells
CSN5: Constitutive photomorphogenic homolog subunit 5
CTC: Circulating tumor cells
CXCR4: Chemokine (C-X-C motif) receptor 4
CYFIP1: Cytoplasmic FMR1 interacting protein 1
CYR61/CCN1: Cellular macromolecular protein cysteine-rich protein 61
DCBLD2: CUB and LCCL domain containing 2
DDR2: Discoidin domain receptor tyrosine kinase 2
DHA: Dihydroartemisinin
DR: Diabetic retinopathy
EBs: Embryoid bodies
ECM: Extracellular matrix
ECs: Endothelial cells
EGFR: Epidermal growth factor receptor
EMT: Epithelial mesenchymal transition
ENT1: Equilibrative nucleoside transporter 1
ERK: Extracellular regulated protein kinases
ERM: Ezrin/Radixin/Moesin
ESA: Essential specific antigen
*ESCC*: Esophageal squamous cell carcinoma
EZH2: Enhancer of zeste homolog 2
FAK: Focal adhesion kinase
FHOD3: FH1/FH2 domain-containing protein 3
FN1: Fibronectin
FOXM1: Forkhead box M1
FOXO3a: Forkhead box O3a
FRK: Fyn-related kinase
FTO: Fat mass and obesity-associated protein
FxOH: Fucoxanthinol
GAL3: Galactose lectin 3
GC: Gastric cancer
GlcNAc: N-acetyl-D-glucosamine
GRB2: Growth factor receptor-bound protein 2
HACE1: HECT domain and ankyrin repeat containing, E3 ubiquitin protein ligase 1
HBx: HBV X protein
HCC: Gepatocarcinoma
HDAC4: Gistone deacetylase 4
HER2: Human epidermal growth factor receptor-2
HIF1-α: Hypoxia inducible factor-1
HLA-B: Human leukocyte antigen class I-B
ICAM1: Intercellular cell adhesion molecule-1
ICH: Intracerebral hemorrhage
I-EGF: Integrin epidermal growth factor-like
IER2: Immediate early response protein 2
IGF-1R: Insulin-like growth factor 1 receptor
IKK: IB kinase
ITGB1: Integrin β-1
JNK: C-Jun N-terminal kinase
KAT1: Lysine acetyltransferase 1
KLF4: Kruppel-like factor 4
LAMC2: Laminin, gamma 2
LC: Lung cancer
LDV: Leu-Asp-Val
LMTK3: Lemur tyrosine kinase 3
MAPK: Mitogen-activated protein kinase
MarvelD1: MAL and related proteins for vesicle trafficking and membrane link domain containing 1
METTL3: Methyltransferase like 3
MFAP2: Microfibrillar-associated protein 2
MGAT3: Mannosyl (beta-1,4-)-glycoprotein beta-1,4-Nacetylglucosaminyltransferase
MM: Multiple myeloma
MMP-2: Matrix metalloproteinase-2
MMP-9: Matrix metalloproteinase-9
MUC20: Mucin 20
MS: Multiple sclerosis
NCKAP1: NCK-associated protein 1
NF-κB: Nuclear factor kappa-B
NRP1: Neuropilin-1
OC: Ovarian cancer
p-AKT: Phosphorylated serine/threonine kinase
PC: Prostate cancer
PCDHB9: Protocadherin Beta 9
PDAC: Pancreatic ductal adenocarcinoma
PDGFR: Platelet-derived growth factor receptor
PECAM-1 or CD31: Platelet endothelial cell adhesion molecule-1
p-eNOS: Phosphorylated endothelial-type nitric oxide synthase
PFK: Phosphatidylinositol
PI3K: Phosphatidylinositol 3-kinase
POSTN: Periostin, osteoblast specific factor
PPAR: Peroxisome proliferator-activated receptor
prEC: Prostate epithelial cells
PSCs: Pancreatic stellate cells
PSI: Plexin-sempahorin-integrin
PTCs: Proximal tubular cells
PXN: Paxillin
RAC1: Ras-related C3 botulinum toxin substrate 1
RAC1: Ras-related C3 botulinum toxin substrate 1
Rap1: RAP1 GTPase activating protein
RBP2: Retinol binding protein 2
RC: Renal cancer
RCP: Rab-coupling protein
RGD: Arg-Gly-Asp
rMCs: Retinal Müller cells
RMECs: Retinal microvascular endothelial cells
ROCK: Rho associated coiled-coil containing protein kinase
ROS: Reactive oxygen species
SATB1: Special AT-rich sequence binding protein 1
SCC: Squamous cell carcinoma
SDC1: Syndecan 1
SERPINB5: Serpin peptidase inhibitor, group B, member 5
sEV: Small extracellular vesicle
SFK: Src family kinases
SIPA1: Signal-induced proliferation- associated gene 1
SOX2: SRY-box containing gene 2
SOX9: SRY-box containing gene 9
STAT1: Signal transducer and activator of transcription 1
T-ALL: T acute lymphoblastic leukemia
TF/FVIIa: Coagulation initiation protein
TFEB: Transcription factor EB
TG2: Transglutaminase 2
TGF-β: Transforming growth factor β
THBS4: Thrombospondin 4
TIMP1: Matrix metalloproteinase inhibitor 1
TMDs: Transmembrane helical domains
TME: Tumor microenvironment
TN-C: Tenascin-C
TRIM11: Tripartite motif-containing 11
TNBC: Triple-negative breast cancer
VCAM-1: Vascular cell adhesion molecule 1
VEGF: Vascular endothelial growth factor
VEGFR: Vascular endothelial growth factor receptor
VM: Vasculogenic mimicry
VSMC: Vascular smooth muscle cells
YTHDF2: YTH domain family member 2
YWHAZ: 14–3-3-zeta
YY1: Yin Yang 1
ZEB1: Zinc Finger E-Box Binding Homeobox 1
ZIP4: Zrt- and Irt-like protein 4
ZNF304: Zinc finger transcription factor
ZNF750: Zinc finger protein 750
